# Antitumor Activity of *Warbugia ugandensis*: Methanolic Extracts and Gene Regulation in Colorectal Cancer

**DOI:** 10.3390/nu17030471

**Published:** 2025-01-28

**Authors:** John M. Macharia, John K. Maina, Afshin Zand, Betsy Rono Cheriro, Tímea Varjas, Dávid Sipos, Zsolt Káposztás, Ferenc Budán, Orsolya Liza Kövesdi, Bence L. Raposa

**Affiliations:** 1 Doctoral School of Health Sciences, Faculty of Health Science, University of Pẻcs, 7621 Pécs, Hungary; 2 Department of Animal Science, Faculty of Agricultural and Food Sciences and Environmental Management, University of Debrecen, 4032 Debrecen, Hungary; johnkiguru97@gmail.com; 3 Department of Public Health Medicine, Medical School, University of Pécs, 7624 Pécs, Hungary; afshin.zand@aok.pte.hu (A.Z.); timea.varjas@aok.pte.hu (T.V.); 4 Department of Health Records and Information Management, School of Public Health, College of Health Science, Jomo Kenyatta University of Agriculture and Technology, Nairobi 43844-00100, Kenya; bcheriro@yahoo.com; 5 Institute of Diagnostics, Faculty of Health Sciences, University of Pécs, 7621 Pécs, Hungary; 6 Faculty of Health Sciences, University of Pécs, 7621 Pécs, Hungary; 7 Institute of Physiology, Medical School, University of Pécs, 7624 Pécs, Hungary; 8 Institute of Basics of Health Sciences, Midwifery and Health Visiting, Faculty of Health Sciences, University of Pécs, 7621 Pécs, Hungary

**Keywords:** *Warbugia ugandensis*, colorectal cancer, phytotherapeutics, genome responses, methanolic extracts, regulatory pathways

## Abstract

A promising approach to accelerating the development of innovative anti-cancer therapies involves the evaluation of natural plant compounds. In this study, we focused on examining the effects of *Warbugia ugandensis* (*W. ugandensis*) methanolic root and stem infusions on the activity of five target genes—*COX-2*, *CASPS-9*, *Bcl-xL*, *Bcl2*, and *5-LOX*—using colorectal cancer (CRC) cell lines (Caco-2). The plant extracts were prepared for testing by dissolving them in dimethyl sulfoxide (DMSO) after undergoing a step-by-step extraction process. Caco-2 cells were then treated with different concentrations of the extracts, and RNA was extracted and purified for analysis. Our results demonstrated a dose-dependent relationship between the phytoconstituents of *W. ugandensis* and the overexpression of *CASP9*, along with the downregulation of *COX-2*, *5-LOX*, *Bcl-xL*, and *Bcl2* genes. This suggests that *W. ugandensis* acts as a potent natural inhibitor of CRC progression. Given the potential clinical benefits, we propose the use of *W. ugandensis* methanolic root and stem extracts as promising organic inhibitors for CRC tumorigenesis, with more in vitro studies warranted to validate and expand on our findings. Additionally, we recommend further studies to identify and characterize the specific metabolites in *W. ugandensis* that contribute to the modulation of pathways responsible for inhibiting CRC growth.

## 1. Introduction

### 1.1. Etiology of Colorectal Cancer and Its Associated Carcinogenesis

Colorectal cancer (CRC) remains a complex and prevalent malignancy, with its pathophysiology intricately shaped by a combination of genetic, lifestyle, and environmental factors [1]. Although our understanding has progressed, numerous aspects of CRC development remain to be fully elucidated, highlighting the disease’s multifactorial nature. In particular, socioeconomic disparities and lifestyle factors—including diet, physical inactivity, and exposure to various environmental carcinogens—contribute substantially to CRC incidence, especially in developing countries where the burden of CRC is increasing at an alarming rate. The genetic landscape of CRC reveals significant insights, involving both inherited and acquired genetic mutations that drive disease progression [1,2].

Landmark research has shed light on the transformation of normal colorectal epithelium into adenomatous polyps, often considered precursors to malignancy, and the subsequent progression to invasive cancer. Key molecular pathways, such as the Wnt/β-catenin, Ras/Raf/MEK/ERK, and PI3K/AKT pathways, play central roles in CRC pathogenesis, influencing cellular proliferation, apoptosis, and differentiation [1,2,3]. Despite these advances, the precise interactions among genetic, epigenetic, and environmental contributors in CRC remain partially understood, necessitating further research to uncover targeted therapeutic approaches and effective preventive strategies [2].

CRC stands out as the most prevalent among gastrointestinal tumors. The tumor microenvironment plays a vital role in the process of tumor development. Colorectal cancer (CRC) is the third most commonly diagnosed cancer globally and the second leading cause of cancer-related deaths, with nearly 70% of these deaths occurring in less developed regions [4]. In 2020, cancer-related deaths were approximately 9.96 million, with projections estimating this number could rise to 16.3 million by 2040CRC develops through three main pathways: the adenoma-carcinoma sequence, the serrated pathway, and the inflammatory pathway, which contribute to its heterogeneous nature based on tumor location and molecular alterations. Chronic inflammation in the colon can facilitate carcinogenesis by inducing oxidative stress, leading to DNA damage and the initiation of tumors [5].

There has been notable progress in comprehending the molecular mechanisms behind adenomatous polyps and the development of cancer [2]. Among gastrointestinal tumors, CRC has the highest incidence. The tumor microenvironment (TME) plays a critical role in the development and progression of colorectal cancer (CRC). The TME consists of a dynamic and complex network of cancer cells, stromal cells, immune cells, and extracellular matrix components, all of which interact to promote tumorigenesis. These interactions influence key processes such as inflammation, angiogenesis, immune evasion, and metastasis. For instance, cancer-associated fibroblasts (CAFs) and tumor-infiltrating immune cells, such as macrophages and T-cells, release cytokines, chemokines, and growth factors that drive tumor growth and alter the immune response. Furthermore, hypoxic conditions within the TME can enhance tumor invasiveness and resistance to therapies. Targeting the TME has become a promising approach in CRC treatment, as it holds potential to disrupt these tumor-promoting processes [4]. Chronic inflammation in the colon can promote cancer development by inducing oxidative stress, which leads to DNA damage and the initiation of tumor growth [5].

The global incidence of colorectal cancer (CRC) shows significant variation, with Western countries reporting the highest prevalence, while sub-Saharan Africa experiences the lowest rates [6]. CRC is a disease that primarily affects industrialized nations with a western way of life.

Cancer development is strongly shaped by factors influencing life expectancy, including lifestyle choices (such as smoking, obesity, and exercise) and social determinants (such as education, income, and public health expenditure). The development of cancer prevention and treatment techniques must take life expectancy levels into account [7]. Recent research suggests that leading a healthy lifestyle marked by a balanced diet, regular physical activity, maintaining a healthy weight, and limiting alcohol consumption can reduce the risk of CRC by up to 37% [8].

The management of colorectal cancer involves a multidisciplinary approach that integrates surgery, chemotherapy, radiation therapy, immunotherapy, and targeted therapies. Surgical resection remains the cornerstone for early-stage disease, often complemented by adjuvant chemotherapy for high-risk cases. Standard chemotherapy regimens include fluoropyrimidines (e.g., 5-fluorouracil), oxaliplatin, and irinotecan, either as monotherapies or in combination (e.g., FOLFOX or FOLFIRI) [9,10].

For advanced or metastatic colorectal cancer, targeted therapies such as bevacizumab (anti-VEGF) and cetuximab or panitumumab (anti-EGFR) are employed based on tumor molecular profiles. Immunotherapy, particularly immune checkpoint inhibitors (e.g., pembrolizumab, nivolumab), is effective in patients with mismatch repair-deficient (dMMR) or microsatellite instability-high (MSI-H) tumors. Additionally, emerging approaches, such as adoptive cell therapy and vaccines, are under investigation in clinical trials. Lifestyle interventions and chemoprevention with agents like aspirin are also being explored for prevention and recurrence risk reduction. The choice of therapy is tailored to the tumor’s stage, molecular characteristics, and patient factors, ensuring a personalized approach to optimize outcomes [9,10,11,12].

### 1.2. Application of Warburgia Plant Species for Medical Purposes

*Warburgia ugandensis* (W. ugandensis), a notable member of the Canellaceae family and classified within the genus *Warburgia*, has been historically valued in traditional medicine for its versatile medicinal applications [13]. Native to various regions across East Africa, particularly in Uganda, Kenya, and Tanzania, this plant holds a central role in indigenous healthcare practices. Known for its rich phytochemical composition, *W. ugandensis* has been widely recognized for its therapeutic potential, especially in treating a spectrum of infectious and inflammatory conditions. While the powdered bark is most commonly used in traditional formulations, the leaves, roots, and stems are also harnessed in various herbal preparations, providing alternative sources of bioactive compounds and enhancing the plant’s therapeutic applications [14].

Traditionally, *W. ugandensis* has been employed to address numerous ailments, ranging from gastrointestinal issues and respiratory infections to broader infectious diseases, due to its reported antimicrobial, antifungal, and anti-inflammatory properties. Recent research has revealed that the plant contains diverse phytochemicals, such as sesquiterpenoids, flavonoids, and tannins, which are believed to contribute to its broad-spectrum pharmacological effects [15,16]. These bioactive constituents have sparked interest in the scientific community, particularly in light of rising antimicrobial resistance, as *W. ugandensis* represents a promising source of natural compounds with potential for drug development [17]. However, further investigation is needed to isolate specific compounds, assess their bioactivity, and evaluate the plant’s safety and efficacy for modern therapeutic use. By elucidating the pharmacological properties of *W. ugandensis*, ongoing research aims to bridge the gap between traditional knowledge and modern medical applications, paving the way for potential advancements in phytotherapy [15,16]. Although concoctions or treatments of the bark are the most common preparations, snuff or smoke inhalation are frequently used. *W. ugandensis* is frequently used alone, but occasionally, it is mixed with other medicinal herbs to increase the beneficial effects [13].

### 1.3. Cytotoxic Effects of W. ugandensis on Cancer Cell Lines

*W. ugandensis* has demonstrated notable cytotoxic effects on cancer cell lines due to its bioactive compounds, primarily sesquiterpenoids and neolignanamides, which exhibit antiproliferative and pro-apoptotic properties [13]. Studies highlight sesquiterpenoid compounds such as polygodial as significant contributors to the cytotoxic activity. These compounds are effective against various cancer cell lines by inducing apoptosis, disrupting cell membrane integrity, and generating reactive oxygen species. For example, sesquiterpenoids from *W. ugandensis* showed cytotoxicity against breast cancer and colon cancer cell lines, supporting their potential as anticancer agents [18,19]. Sesquiterpenoids have demonstrated significant cytotoxic activity against both breast cancer and colon cancer cell lines. These bioactive compounds, belonging to the terpenoid class of secondary metabolites, exhibit their anticancer potential through mechanisms such as inducing apoptosis, arresting the cell cycle, and modulating key signaling pathways involved in cancer progression. Dysregulation of caspase activity is a hallmark of CRC, contributing to the evasion of programmed cell death. Sesquiterpenoids may restore caspase activation (e.g., caspase-3 and caspase-9), triggering apoptosis in cancer cells. Overexpression of anti-apoptotic proteins like Bcl-2 and Bcl-xL is common in CRC. Sesquiterpenoids could downregulate these proteins while upregulating pro-apoptotic factors like Bax, restoring apoptotic balance. Sesquiterpenoids may disrupt mitochondrial membrane potential in CRC cells, releasing cytochrome c and activating intrinsic apoptosis pathways. PI3K/Akt/mTOR pathway is frequently hyperactivated in CRC, promoting cell survival, proliferation, and resistance to therapy. Sesquiterpenoids can inhibit PI3K or downstream effectors like mTOR, sensitizing CRC cells to apoptosis and reducing tumor growth. Aberrant Wnt signaling is a defining feature of CRC, driven by mutations in APC, β-catenin, or Axin. Sesquiterpenoids might downregulate β-catenin levels or block its nuclear translocation, suppressing transcription of oncogenic targets like MYC and cyclin D1. Hyperactivation of MAPK signaling, often due to KRAS or BRAF mutations, is implicated in CRC. Sesquiterpenoids could inhibit ERK phosphorylation, impairing cancer cell proliferation. Chronic inflammation, a driver of CRC, involves persistent activation of NF-κB. Sesquiterpenoids may inhibit NF-κB, reducing pro-inflammatory cytokines and chemokines that fuel tumor growth. CRC cells often exploit oncogenes (e.g., MYC) or lose tumor suppressors (e.g., p53) to evade normal growth control. Sesquiterpenoids could selectively inhibit oncogene activity or restore p53-mediated responses, leading to targeted cytotoxicity against cancer cells while sparing normal tissues. Studies have highlighted their ability to inhibit the growth of cancer cells selectively, while sparing normal cells, making them promising candidates for further development as targeted anticancer agents [20,21]. Furthermore, the natural origin of these compounds suggests lower toxicity and better biocompatibility compared to conventional chemotherapeutic drugs. Research exploring the synergistic effects of sesquiterpenoids with existing chemotherapies has also shown encouraging results, further supporting their therapeutic potential in combating malignancies such as colorectal and breast cancer [21].

Neolignanamides compounds from *W. ugandensis* were identified to target enzymes like cyclooxygenase-2 (*COX-2*), 5-lipoxygenase (*5-LOX*), and topoisomerases I and II. This activity correlates with their ability to reduce inflammation and inhibit cancer cell proliferation. Specifically, neolignanamides demonstrated strong binding affinity to these enzymes and successfully inhibited cancer cell growth in vitro [19]. The mechanisms of cytotoxic effects include DNA damage, inhibition of key cancer-promoting pathways, and oxidative stress induction, causing cell cycle arrest and inducing apoptosis in cancer cells. [13,18,19].

As reported in our previous study [13], *W. ugandensis* extracts demonstrate selective cytotoxicity, meaning they are more toxic to cancer cells than to normal cells, making it a promising candidate for therapeutic development. Further, we reported that the effectiveness of *W. ugandensis* extracts is concentration-dependent, with higher doses showing greater cytotoxic effects. However, optimal dosages vary across studies and cancer cell lines.

Our research principally aimed at assessing the effects of *W. ugandensis* methanolic root and stem infusions on the level of activity of five desired genes: *COX-2*, *CASPS-9*, *Bcl-xL*, *Bcl2*, and *5-LOX*. This research highlights the potential of *W. ugandensis* as a natural alternative to synthetic inhibitors in the treatment of CRC, despite limited knowledge about its effects, primarily based on in vitro and in vivo studies. It reveals how the plant’s phytochemicals regulate gene expression in CRC, introducing a novel mechanism of action. This study provides an in-depth evaluation of *W. ugandensis* in vitro at various stages, laying the groundwork for future in vivo and clinical applications, while underscoring the effectiveness of methanol as a highly effective extraction solvent.

## 2. Materials and Methods

### 2.1. Sourcing Caco-2 Cell Lines

The Department of Biochemistry and Medical Chemistry at the University of Pécs provided the Department of Public Health with Caco-2 cell lines, which were originally obtained from the American Type Culture Collection (ATCC, Manassas, VA, USA). These cell lines are widely used in toxicological and cancer research, particularly for transfection studies. The Caco-2 cells were cultured according to the manufacturer’s instructions [16].

### 2.2. Plant Extraction Processes and the Locations Where Plants Are Obtained

The selected stem and root organs were shade-dried and finely powdered. Methanol was used as the solvent for serial exhaustive extraction (SEE). For this process, 1000 g of *W. ugandensis* plant material was placed in a flask and extracted with methanol for three days, with frequent shaking. The crude solvent extracts were filtered through Whatman filter paper with particle diameters ranging from 4 to 1 μm to remove chlorophyll. This extraction process was repeated three times to ensure complete recovery of all soluble phytoconstituents [17].

To concentrate the extract, the solvent was evaporated using a rotary evaporator under reduced pressure at a temperature range of 40–50 °C. The aqueous extract was lyophilized using a freeze dryer. The resulting dry, solvent-free metabolites were stored in tightly sealed sample vials, secured with parafilm, and kept in a desiccator at 4 °C in a refrigerator [18].

The plant materials of *W. ugandensis* were collected from Egerton University, located in the eastern Mau water catchment area in Njoro, Kenya. The collection site is positioned at 0°22′11.0′′ S, 35°55′58.0′′ E (latitude: −0.369734, longitude: 35.932779) at an elevation of 2238 m above sea level [19].

Methanol is widely used as an extraction solvent for plant metabolites because of its excellent ability to dissolve a broad range of polar and moderately non-polar compounds, including alkaloids, phenolics, flavonoids, and glycosides. Its high polarity allows it to efficiently penetrate plant cell walls and extract bioactive components. Additionally, methanol is relatively inexpensive, easy to handle, and has a low boiling point, making it simple to remove during the concentration step. These characteristics make methanol an effective and practical choice for extracting diverse phytochemicals from plant material [22,23,24,25].

### 2.3. DMSO as a Tool for Biological Evaluation

Dimethyl sulfoxide (DMSO) is a versatile solvent widely utilized in toxicology and pharmacology for dissolving various drugs, enhancing drug delivery, and solubilizing plant extracts [20]. In this study, DMSO was used as both a suspending medium and an inert diluent for crude plant extracts that were insoluble in water. A 30 mg/mL stock solution was prepared by dissolving the extracts in 0.5% DMSO, with double-distilled phosphate-buffered saline (ddPBS) serving as the diluent solvent. The stock solution was subsequently diluted to obtain final concentrations of 2 mg/mL, 1 mg/mL, and 0.5 mg/mL for treating Caco-2 cell lines.

### 2.4. Passaging Caco-2 Cell Lines for Extract Exposure

The Petri dish or flask containing Caco-2 cells was carefully placed inside a laminar flow hood to maintain sterility. After opening the dish or flask, the spent culture medium was aspirated, and the cells were washed twice with phosphate-buffered saline (PBS). PBS containing EDTA was then added, allowed to sit briefly, and subsequently aspirated gently. To facilitate cell detachment from clumps and the culture surface, 2 mL of trypsin was applied and distributed evenly by gently tilting the dish side-to-side. The dish was incubated in a thermostat for 5 min.

Following incubation, the dish was removed, and once visible detachment of cells was observed, fresh Caco-2 medium was added to neutralize the trypsin. Since Caco-2 cells are adherent, the contents of the dish were transferred into a tube and centrifuged at 125 rpm for 5 min. The supernatant was aspirated, leaving the Caco-2 cell pellet at the bottom of the tube. Fresh medium was added, and the cells were resuspended gently using a pipette.

The cell suspension was distributed into new culture dishes, fresh medium was added, and the dishes were placed in a thermostat for further growth. The confluence of the cell cultures was monitored until it reached 70–80%, at which point the cells were ready for treatment.

### 2.5. Caco-2 Cell Lines Treated with Solutions Containing Plant Extracts

After replenishing the aged Caco-2 cell lines with fresh Caco-2 medium, 200 μL of extract solutions at varying concentrations (0.5 mg/mL, 1 mg/mL, and 2 mg/mL) were administered to the cells in 6-well plates (surface area ~9.6 cm^2^/well), Seed 200,000–300,000 cells per well in 2 mL of medium. The treated cells were then incubated at 37 °C for 36 h. Following the incubation period, the condition of the cells was assessed under a light microscope. Preliminary dose- and time-dependent studies informed the experimental design, and gene expression analysis was a critical component of our investigation into the molecular effects of the plant’s compounds. The application of 0.5, 1, and 2 mg/mL concentrations in RT-PCR experiments served a critical purpose in evaluating the dose–response relationship, which is essential for understanding how gene expression levels vary with increasing treatment doses. By testing multiple concentrations, identification of the optimal treatment concentration (the dose that maximizes the desired gene expression effects without inducing cytotoxicity or off-target effects) could be determined. Moreover, this approach was designed to ensure the sensitivity of the assay, meaning that it could reliably detect even subtle changes in gene expression, and its specificity, ensuring the changes observed are directly attributable to the treatment and not confounded by background noise or non-specific interactions. Finally, selecting these concentrations was aimed at mimicking physiologically relevant conditions, ensuring that the observed effects are representative of what might occur in vivo, thereby enhancing the translational relevance of the findings.

The 36–48 h exposure period for colorectal cancer cell lines, such as Caco-2, is crucial for allowing treatments to interact with cells across the cell cycle, ensuring sufficient time for measurable effects like apoptosis, DNA damage, or gene expression changes. The duration was chosen based on prior observations of gene expression changes and cell viability assays conducted during the initial optimization phase of the study. In addition, this duration aligns with the cell doubling time, providing reliable assay results while avoiding over-confluence or nutrient depletion, making it ideal for evaluating cytotoxic or cytostatic effects of therapeutic agents. Treatments were applied sequentially, starting from the lowest concentration (0.5 mg/mL) and progressing to the highest concentration (2 mg/mL), to evaluate the dose- and time-dependent responsiveness of the cells. This approach facilitated the optimal detection and assessment of the modulatory effects induced by increasing concentrations of the extract. Cell growth and potential biological effects were monitored at 12 h intervals throughout the treatment period. The purpose of this monitoring was to complement our RT-PCR analysis by identifying the time frame during which the most pronounced biological responses occurred. Gene expression analysis was conducted in parallel to assess the molecular effects at key time points identified during this interval monitoring. Essentially, the 12 h interval is essential in order to capture dynamic time-dependent effects on cell growth, gene expression, and other biological responses, providing insight into the kinetics of treatment effects.

### 2.6. RNA Extraction

After removing the media from the cell cultures, the cells were washed twice with PBS and treated with trypsin-EDTA to detach them. The resulting cell suspension was transferred into a 4 cm^3^ centrifuge tube. To begin RNA extraction, 1 cm^3^ of ExtraZol Tri-reagent Solution (Thermo Fisher Scientific Inc., Waltham, MA, USA) was added, and the mixture was incubated at room temperature for 5 min. Subsequently, 0.2 cm^3^ of chloroform was added, followed by a brief 2–3 min incubation. The sample was centrifuged at 12,000× *g* for 10 min at 2–8 °C.

Following centrifugation, the aqueous phase was carefully transferred to a clean tube. To precipitate RNA, 0.2 cm^3^ of isopropyl alcohol was added, and the mixture was incubated for 10 min before being centrifuged again at 12,000× *g* for 10 min at 2–8 °C. Although the RNA precipitate is often invisible prior to centrifugation, a gel-like pellet formed at the bottom of the tube upon centrifugation.

The supernatant was discarded, and the RNA pellet was washed with 1 cm^3^ of 75% ethanol. The mixture was vortexed briefly and centrifuged at 7500× *g* for 5 min at 2–8 °C. After removing the ethanol, the pellet was allowed to dry and was then re-suspended in 50–100 µL of RNase-free, DEPC-treated water. The re-suspended RNA was incubated at 55 °C for 10 min with periodic vortexing to ensure complete dissolution. The extracted total RNA was stored at −80 °C until further use.

### 2.7. RNA Concentration and Purity Assessment Using UV Spectroscopy

UV spectroscopy was utilized to measure the concentration and purity of RNA. To improve the accuracy of the analysis, RNA samples were pre-treated with RNase-free DNase to remove any DNA contamination. Residual impurities, such as proteins and phenols, which could interfere with absorbance measurements, were meticulously eliminated during sample preparation.

The absorbance of a diluted RNA sample was measured at 260 nm and 280 nm. Nucleic acid concentration was calculated using the Beer-Lambert law, which establishes a direct relationship between absorbance and concentration. The purity of the RNA was evaluated based on the A260/A280 ratio, with values between 1.8 and 2.1 considered indicative of high purity.

### 2.8. SYBR Green qRT-PCR Method: Equipment and Step-by-Step Protocol

Real-time quantitative PCR (qPCR) enables precise nucleic acid quantification with high sensitivity, specificity, and reproducibility. The One-Step Detect SyGreen Lo-ROX RT-PCR kit (Nucleotest Bio Ltd., Budapest, Hungary) was employed to perform one-step PCR, which integrates reverse transcription and amplification, on a 96-well plate using the LightCycler 480 qPCR platform (Hoffmann-La Roche, Basel, Switzerland), following the manufacturer’s protocol.

The thermal cycling program was configured as follows:

1. Incubation at 42 °C for 5 min.
2. Denaturation at 95 °C for 3 min.
3. Forty-five amplification cycles, each consisting of the following:
  - A temperature of 95 °C for 5 s;
  - A temperature of 56 °C for 15 s;
  - A temperature of 72 °C for 5 s.

Fluorescence readouts were recorded at the end of each cycle. To confirm amplification specificity, melting curve analysis was conducted after the amplification process, with the program set at 95 °C for 5 s, 65 °C for 60 s, and 97 °C indefinitely.

The reaction mix consisted of a total volume of 20 μL, prepared as follows:

- A 5 μL mRNA template supplemented with sterile double-distilled water;
- A 10 μL Master Mix;
- A 0.4 μL RT Mix;
- A 0.4 μL dUTP;
- Primers of 0.4 μL.

Primers were designed using Primer Express software 3.0 (Thermo Fisher Scientific Inc., Waltham, MA, USA) and synthesized by Integrated DNA Technologies (Coralville, IA, USA). Details of the primer sequences are provided in Table 1.

### 2.9. Analysis of qRT-PCR Results

The PCR results were expressed using Cp (crossing point) values, representing the intersection of the amplification curve with the threshold line. Fold changes in the expression of target genes relative to the control sample were calculated using the 2^−ΔΔCp^ method (Livak technique) [21]. It is significant to note that 2^−ΔΔCp^ is the standard notation in Livak’s method for calculating relative gene expression. The housekeeping gene *HPRT1* was employed as the internal control to normalize the data and ensure consistency across the experimental study.

### 2.10. Data Analysis

Statistical analysis was performed using IBM SPSS Statistics for Windows, Version 26.0.3 (IBM Corp., Armonk, NY, USA, 2019) and Microsoft Excel (Microsoft Corp., 2013, Redmond, WA, USA). Normality of the data was assessed using the Kolmogorov–Smirnov test. Following this, comparisons of mean values for the relevant variables were conducted using ANOVA with post hoc analysis. Results were considered statistically significant if the *p*-value was ≤0.05 at a 95% confidence interval.

## 3. Results

### 3.1. Regulation of COX-2 Gene Expression Following Administration of Methanolic Root and Stem Extracts at Increasing Dosage Concentrations

**Figure 1:**
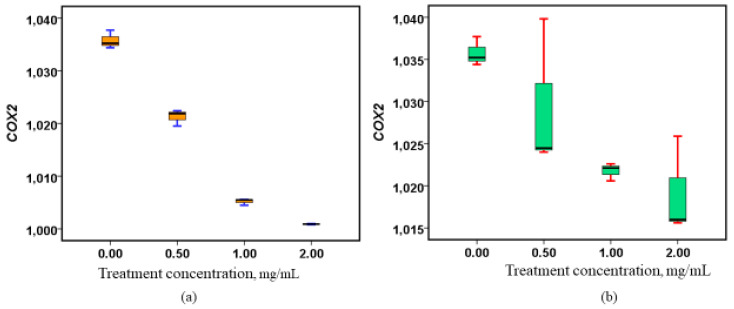
Alterations in *COX-2* gene expression subsequent to treatment with methanolic stem and root extracts. (**a**) Box plot showing the regulatory properties of the stem extracts on *COX2*, (*p* = 0.001), (**b**) Box plot showing the regulatory properties of the root extracts on *COX2*, (*p* = 0.021).

The treatment of Caco-2 cell lines with methanolic extracts from both the roots and stems of the plant resulted in a marked, dose-dependent reduction in COX-2 gene expression (Figure 1). As the concentration of the extracts increased, a progressive downregulation of *COX-2* expression was observed, suggesting a potential inhibitory effect on the gene’s activity. Statistical analysis revealed that the effects of the root extracts were significant (*p* = 0.021), while the stem extracts showed even stronger significance (*p* = 0.001), indicating that both extracts have a substantial impact on *COX-2* gene regulation. The results demonstrate that both root and stem extracts significantly influence COX-2 gene regulation, with the stem extracts showing a more pronounced effect. This suggests that the bioactive compounds present in these extracts may have a strong potential to modulate inflammation, as COX-2 is a key enzyme involved in the inflammatory response. The findings highlight the therapeutic relevance of these extracts, potentially positioning them as candidates for developing anti-inflammatory treatments or as complementary agents in managing conditions where *COX-2* plays a critical role (Figure 1).

### 3.2. Regulation of CASP9 Gene Expression Following Administration of Methanolic Root and Stem Extracts at Increasing Dosage Concentrations

**Figure 2:**
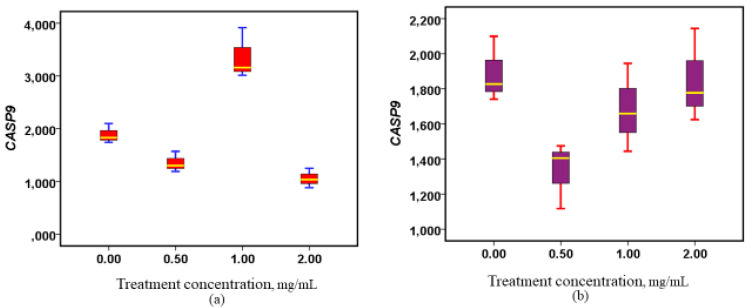
Modulation of *CASP9* gene expression following exposure to methanolic stem and root extracts. (**a**) Box plot showing the regulatory properties of the stem extracts on *CASP9*, (*p* = 0.001), (**b**) Box plot showing the regulatory properties of the root extracts on *CASP9*, (*p* = 0.059).

Upon treatment of Caco-2 cell lines with methanolic extracts from the roots and stems of W. ugandensis, a dose-dependent increase in CASP9 gene expression was observed in response to the root extracts (Figure 2). Notably, this upregulation was evident at all concentrations of the root extracts, with the highest levels of activity observed at the greater doses. Conversely, the stem extracts exhibited a more variable response. While the stem extracts did show an increase in *CASP9* gene expression, the upregulatory effects were only significant at lower doses. This differential response between the root and stem extracts indicates that the roots may have a more consistent effect across varying concentrations. Statistical analysis revealed that the upregulatory effects in the root extracts were not statistically significant (*p* = 0.059), suggesting a trend toward increased expression that did not reach the threshold for significance. In contrast, the stem extracts showed significant upregulation of *CASP9* gene expression (*p* = 0.001), particularly at lower doses. Additionally, the root extracts demonstrated the strongest upregulatory efficacy at higher doses, further emphasizing the dose-dependent nature of their impact on *CASP9* gene expression (Figure 2).

### 3.3. Regulation of Bcl-xL Gene Expression Following Administration of Methanolic Root and Stem Extracts at Increasing Dosage Concentrations

**Figure 3:**
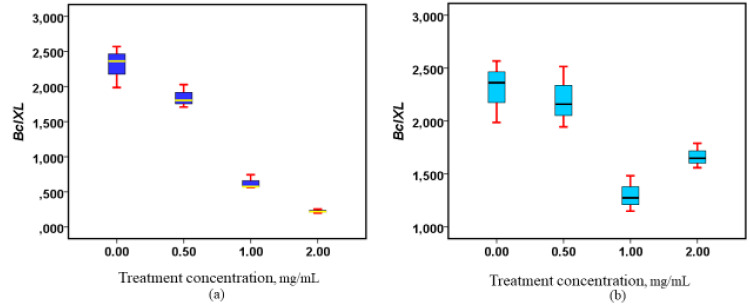
Modulation of *Bcl-xL* gene expression following exposure to methanolic stem and root extracts. (**a**) Box plot showing the regulatory properties of the stem extracts on Bcl-xL, (*p* = 0.001), (**b**) Box plot showing the regulatory properties of the root extracts on Bcl-xL, (*p* = 0.002).

Methanolic extracts from the roots and stems of *W. ugandensis* were applied to Caco-2 cell lines to assess their impact on *Bcl-xL* gene expression. In both root and stem extracts, a clear dose-dependent downregulation of *Bcl-xL* gene expression was observed (Figure 3), suggesting that these extracts may have inhibitory effects on the expression of this anti-apoptotic gene. While the response was generally consistent, an interesting observation was made at the high dose (2 mg/mL) of the root extract, where a slight enhancement in *Bcl-xL* expression was noted, indicating that the effect of the root extract may be more nuanced at higher concentrations. Despite this modest increase at the higher dose in the root extract, both the root and stem extracts induced statistically significant downregulation of *Bcl-xL* expression (*p* = 0.002 for roots and *p* = 0.001 for stems), underscoring the robustness of the extracts’ effects. These findings highlight the potential of *W. ugandensis* extracts, especially at higher concentrations, to modulate gene expression in a dose-dependent manner, with both root and stem extracts exhibiting notable effects on *Bcl-xL* gene regulation. For further details on the dose-dependent changes in expression, refer to Figure 3.

### 3.4. Regulation of Bcl2 Gene Expression Following Administration of Methanolic Root and Stem Extracts at Increasing Dosage Concentrations

**Figure 4:**
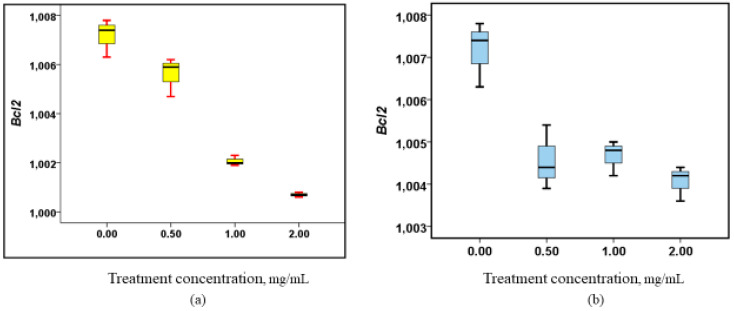
Alterations in *Bcl2* gene expression upon treatment with methanolic root and stem extracts. (**a**) Box plot showing the regulatory properties of the stem extracts on Bcl2, (*p* = 0.001), (**b**) Box plot showing the regulatory properties of the root extracts on Bcl2, (*p* = 0.002).

Both the root and stem extracts of *W. ugandensis* were tested for their effects on *Bcl2* gene expression in Caco-2 cell lines, and both extracts demonstrated a dose-dependent downregulation of *Bcl2* expression (Figure 4). This indicates that both extracts, at varying concentrations, tend to reduce the expression of *Bcl2*, a gene critical for the regulation of apoptosis. Interestingly, when the root extracts were administered at an increased dose of 1 mg/mL, a modest increase in *Bcl2* expression was observed, suggesting a potential shift in gene regulation at this intermediate concentration. However, at a higher dose of 2 mg/mL, this enhanced expression was followed by a return to decreased expression, reinforcing the idea that the response to the root extract may be concentration-dependent. This complex response pattern in root extracts, where the expression initially increases before declining again at higher doses, highlights the nuanced effects of the extract. Despite this fluctuation in the root extracts, both root and stem extracts showed statistically significant changes in *Bcl2* expression (*p* = 0.002 for roots and *p* = 0.001 for stems). These results underline the significant, albeit dose-dependent, influence of *W. ugandensis* extracts on *Bcl2* gene regulation. For a visual representation of these expression patterns, please refer to Figure 4.

### 3.5. Regulation of 5-LOX Gene Expression Following Administration of Methanolic Root and Stem Extracts at Varying Dosage Concentrations

**Figure 5:**
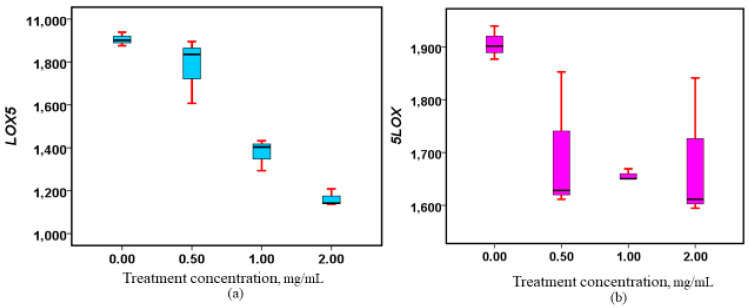
Alterations in *5-LOX* gene expression subsequent to treatment with methanolic stem and root extracts. (**a**) Box plot showing the regulatory properties of the stem extracts on *5LOX*, (*p* = 0.001), (**b**) Box plot showing the regulatory properties of the root extracts on *5LOX*, (*p* = 0.048).

The expression of *5-LOX* genes was reduced in a dose-dependent manner in Caco-2 cell lines treated with methanolic extracts from both the roots and stems of *W. ugandensis* (Figure 5). This indicates that both extracts have the potential to inhibit the expression of the *5-LOX* gene, which is crucial in inflammatory pathways. However, when comparing the effects of the root and stem extracts, the stem extracts displayed a more pronounced and consistent downregulation of *5-LOX* expression, suggesting a stronger effect on gene modulation. In contrast, the root extracts exhibited a slightly different trend, with a modest increase in gene expression at higher concentrations. This indicates that, while the root extracts generally exert a downregulatory effect, they may also induce some gene-promoting activity at elevated doses, highlighting the complexity of their biological impact. Despite these variations, both root and stem extracts induced statistically significant changes in *5-LOX* gene expression, with the root extract yielding a *p*-value of 0.048 and the stem extract showing a highly significant *p*-value of 0.001. These findings underscore the potential of both *W. ugandensis* extracts to modulate gene expression in a dose-dependent manner, with the stem extracts showing superior efficacy in downregulating *5-LOX* expression. For further details on these results, please refer to Figure 5.

**Figure 6:**
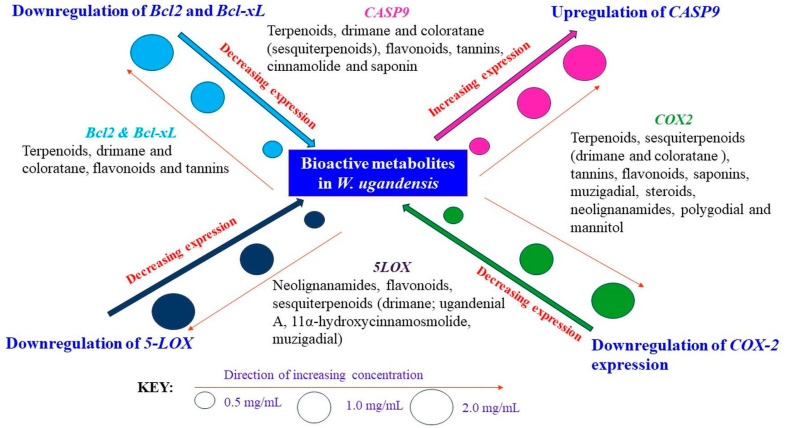
A schematic illustration of the bioactive phytocomponents present in *W. ugandensis*, and their regulatory characteristics on gene targets: *Bcl2*, *Bcl-xL*, *CASP9*, *COX-2* and *5-LOX*.

Figure 6 provides a comprehensive schematic representation that summarizes the potent metabolites found in *W. ugandensis* and their regulatory effects on key gene targets, including *Bcl2*, *Bcl-xL*, *CASP9*, *COX-2*, and *5-LOX*. This illustration encapsulates the diverse bioactive compounds present in the plant and their potential mechanisms of action, highlighting how these metabolites influence gene expression across different cellular pathways. By visualizing the interactions of these metabolites with the specified gene targets, Figure 6 offers a clear overview of the plant’s molecular influence, emphasizing its role in modulating critical genes involved in apoptosis, inflammation, and cell survival. The schematic not only provides insight into the bioactive compounds but also serves as a guide for understanding their complex regulatory activities, which could have significant implications for therapeutic applications. For a detailed summary of these metabolites and their effects on the gene targets, please refer to the figure below.

## 4. Discussion

### 4.1. Modulation of COX-2 Expression by Root and Stem Extracts in Phytotherapy

Cyclooxygenase-2 (COX-2) is an enzyme that typically exhibits low expression in normal physiological conditions but can be markedly upregulated in response to various stress stimuli, such as cytokines, growth factors, tumor necrosis factors, and lipopolysaccharides [2]. The COX-2 enzyme is encoded by the *PTGS2* gene (prostaglandin-endoperoxide synthase 2), commonly referred to as the *COX-2* gene. *COX-2* gene expression refers to the transcription and translation processes by which this gene is activated to produce the COX-2 enzyme [26]. In the context of the colon, these inflammatory signals activate *COX-2* genes, leading to the increased production of prostaglandins that promote inflammation, cellular proliferation, and angiogenesis. Elevated levels of COX-2 enzymes have been strongly linked to the development and progression of colorectal cancer, as the enzyme’s activity enhances cell survival and supports a tumor-friendly microenvironment. This overexpression of *COX-2* not only facilitates tumor cell growth but also aids in the invasion and spread of cancer cells. Consequently, *COX-2* has become a target of interest for therapeutic interventions, as inhibiting its activity may help to mitigate tumor development and improve outcomes in colorectal cancer [1,2,27,28].

Our study demonstrated that methanolic extracts from the roots and stems of *W. ugandensis* significantly decreased *COX-2* gene expression in Caco-2 cell lines, with the effect observed in a dose-dependent manner. This considerable reduction in *COX-2* levels is attributed to the diverse range of bioactive compounds present in the roots and stems of *W. ugandensis*, which may include terpenoids, sesquiterpenoids, tannins, flavonoids, saponins, muzigadial, steroids, polygodial, mannitol, and other anti-inflammatory phytochemicals [9]. These compounds likely interfere with the inflammatory pathways that lead to *COX-2* upregulation, thereby mitigating *COX-2* expression at the molecular level. Such findings highlight the potential of *W. ugandensis* extracts as a natural source of anti-inflammatory agents with promising implications for colorectal cancer therapies, particularly in reducing inflammation-driven cancer progression.

This research underscores the therapeutic potential of *W. ugandensis*, opening avenues for further studies to isolate specific bioactive components responsible for *COX-2* inhibition and explore their mechanisms of action in cancer treatment [29,30,31,32,33,34,35,36,37]. The concentration of metabolites, particularly drimane and coloratane sesquiterpenoids, has been reported to be abundant in the stems [34], which may account for the significant efficacy observed in our results with stem extracts. Notably, both the roots and stems of this valuable plant could therefore serve as promising alternative phytotherapeutic *COX-2* inhibitors, offering a natural option compared to synthetic agents that are commonly associated with adverse effects [1].

### 4.2. Modulation of CASP9 Expression by Root and Stem Extracts in Phytotherapeutic Contexts

*CASP9*, the initiator of the mitochondrial caspase pathway, is a crucial mediator in the regulation of apoptosis, as highlighted in our previous work [9]. Understanding caspase signaling is essential for selectively modulating apoptosis for therapeutic purposes [35,36]. Apoptosis is a vital physiological process that ensures the selective elimination of cells during various biological events [37]. Suppressing spontaneous apoptosis has been linked to an increased risk of cancer [38,39], while a reduced rate of apoptosis has been associated with a higher incidence of colorectal adenoma [40].

Our results demonstrated variable upregulation of *CASP9* in Caco-2 cells when exposed to root and stem extracts, positioning *W. ugandensis* as a promising stimulant of apoptotic effects. Upon treatment with methanolic root and stem extracts, *CASP9* gene expression was upregulated in a dose-dependent manner, with the most significant effects observed at higher concentrations of both extracts. Notably, root extracts showed a more pronounced upregulation of *CASP9* compared to stem extracts.

These results suggest a nuanced, dose-dependent effect of the root and stem extracts on *CASP9* gene expression. The root extracts showed a trend toward increasing *CASP9* expression (*p* = 0.059), but this did not reach statistical significance, meaning that while there might be a potential effect, the data do not provide strong enough evidence to confirm a reliable upregulation at the tested doses. This suggests that the effect might require further investigation with larger sample sizes or a wider range of doses to clarify the trend.

In contrast, the stem extracts demonstrated significant upregulation of *CASP9* gene expression (*p* = 0.001), particularly at lower doses. *CASP9* is a gene involved in apoptosis, or programmed cell death, and its upregulation could have implications for promoting cell death in diseased or abnormal cells. The fact that stem extracts were particularly effective at lower doses highlights their potent action at a potentially more efficient concentration, offering insight into their potential therapeutic use, especially in contexts where controlled apoptosis is desirable, such as in cancer treatment [38].

Additionally, the observation that the root extracts were most effective at higher doses reinforces the idea that the response to these extracts is dose-dependent, meaning that higher concentrations of the root extracts might be required to achieve the maximum therapeutic benefit in terms of *CASP9* upregulation. This suggests that there might be an optimal dose for the root extracts that warrants further exploration to understand the precise dose–response relationship. Overall, these findings suggest that both extracts have distinct but complementary properties in modulating *CASP9* gene expression. The root extracts may be more effective at higher doses, while the stem extracts show greater efficacy at lower doses, highlighting the importance of dosage in their potential applications for therapies that involve apoptosis regulation [38,39].

One potential strategy for chemoprevention is the induction of apoptosis in gastrointestinal epithelial cells [39]. The suppression of apoptosis has been suggested to promote cancer progression [38,39], and a lower rate of apoptosis has been linked to a higher prevalence of colorectal adenoma [40]. Thus, triggering apoptosis in gastrointestinal epithelial cells represents a potential approach in chemoprevention [39]. Consequently, exploring apoptotic mechanisms presents a viable avenue for colorectal cancer management. The relationship between *CASP9* and colorectal cancer remains poorly understood, and studying its association with clinicopathological features and survival could provide valuable insights for predicting outcomes and guiding treatment strategies. Additionally, natural phytoconstituents commonly found in *W. ugandensis*, such as terpenoids, drimane and coloratane sesquiterpenoids [41], flavonoids [41], tannins [42], cinnamolide and its derivatives [43], and saponins [44,45,46], have been reported to induce apoptosis through the caspase-dependent cascade

### 4.3. Modulation of Bcl-xL and Bcl2 Expression by Root and Stem Extracts

The Bcl-2 protein family, which includes both anti-apoptotic and pro-apoptotic members, is the most well-defined protein family involved in the regulation of apoptotic cell death. Anti-apoptotic members such as Bcl2 and Bcl-xL inhibit apoptosis by either sequestering the proforms of caspases (death-driving cysteine proteases) or preventing the release of mitochondrial apoptogenic factors like cytochrome c and AIF (apoptosis-inducing factor). Once in the cytoplasm, cytochrome c and AIF directly activate caspases, which then cleave various cellular proteins, culminating in apoptosis [37].

Our study showed that stem extracts from *W. ugandensis* significantly downregulated the expression of *Bcl-xL* and *Bcl2*. Both genes exhibited similar modulatory expression patterns, which can be attributed to their shared membership in the anti-apoptotic Bcl-2 family [47]. The decreased expression of *Bcl-xL* and *Bcl2* correlates with enhanced apoptotic effects, a critical factor in the antiproliferative response against cancerous cells. In summary, *Bcl-xL* and *Bcl2* are key mediators of apoptosis [48] and may represent promising therapeutic targets for colorectal cancer (CRC) treatment, as demonstrated by our experimental results. The characteristic downregulation of Bcl-xL and Bcl2 observed with the treatment extracts in our study is likely due to the presence of a variety of apoptosis-stimulating active metabolites in *W. ugandensis*.

The data from this study indicate that the inhibition of *Bcl-2/Bcl-xL* enhances their decreased expression, leading to a reduction in CRC (Caco-2) cell line growth. For the first time, we demonstrate that the modulation of *Bcl2/Bcl-xL* and their associated intracellular signaling pathways plays a pivotal role in the potential anticancer activity of *W. ugandensis* against colorectal cancer cells. Our results show that the root and stem extracts of *W. ugandensis* significantly influence the expression of genes involved in apoptosis, such as *CASP9*, a key regulator of the mitochondrial pathway of apoptosis. The upregulation of *CASP9*, particularly in response to the stem extracts at lower doses, suggests a potential mechanism by which the *Bcl2/Bcl-xL* pathway might be affected, as these proteins are central to regulating mitochondrial integrity and apoptosis.

The observed effects indicate that both the root and stem extracts are capable of modulating apoptotic pathways through the regulation of *Bcl2/Bcl-xL*, with a stronger impact observed in the stem extracts, which might correlate with the differential expression of these proteins. This modulation leads to enhanced apoptotic signaling, providing a rationale for the observed anticancer effects of *W. ugandensis* against CRC cells. The dose-dependent nature of the extracts further underscores the potential of this plant for targeted therapeutic strategies, where higher doses of root extracts show more pronounced effects, while stem extracts could offer efficient low-dose options.

Based on these findings, we recommend the use of methanol as the solvent of choice for the effective extraction of *Bcl-xL* and *Bcl2* modulatory phytoconstituents from this plant. Methanol is particularly efficient in extracting bioactive compounds that could modulate these apoptotic pathways, as evidenced by the stronger apoptotic effects seen with this solvent [39]. The identification and characterization of these bioactive compounds in *W. ugandensis* could pave the way for the development of novel therapeutic agents targeting the Bcl2/Bcl-xL pathway in CRC treatment

### 4.4. Modulation of 5-LOX Gene Expression by Root and Stem Extracts

The link between 5-LOX overexpression and cancer development, though not fully elucidated, is well documented, with increased 5-LOX expression often observed during neoplastic transformation, including in colorectal cancer (CRC) [40]. Elevated 5-LOX activity contributes to tumorigenesis by promoting inflammation, cell survival, and metastasis. Consequently, targeting 5-LOX overexpression has emerged as a promising strategy for cancer prevention and treatment.

The findings from our study demonstrate that methanolic extracts from the roots and stems of *W. ugandensis* significantly downregulated 5-lipoxygenase (*5-LOX*) gene expression, with the stem extracts showing the most pronounced beneficial effects. Interestingly, high concentrations of root extracts slightly increased *5-LOX* gene expression, suggesting a concentration-dependent response that may warrant further investigation. The identification of neolignanamides as the active components responsible for the anti-inflammatory and anti-proliferative properties of *W. ugandensis* [19] further strengthens the argument that these compounds play a crucial role in modulating *5-LOX* activity, thereby contributing to reduced *5-LOX* expression.

Previous studies have shown that the inhibition of *5-LOX* reduces cancer cell proliferation and induces apoptosis through the mitochondrial pathway in both in vitro and in vitro models, supporting the therapeutic potential of *5-LOX* inhibitors [41,42]. These findings highlight the role of natural *5-LOX* inhibitors, such as those found in *W. ugandensis*, as a novel therapeutic approach for preventing or treating CRC, reinforcing the idea that *W. ugandensis* extracts may provide a promising alternative to conventional therapies for managing inflammation-driven cancers like CRC.

## 5. Conclusions

The findings of this study underscore *W. ugandensis* as a promising source of anticancer phytochemicals, particularly in its methanolic extracts from the roots and stems, which exhibit significant modulatory effects on key genes involved in colorectal cancer (CRC) progression. The dose-dependent regulation of *CASP9*, along with the downregulation of crucial oncogenes such as *COX-2*, *5-LOX*, *Bcl-xL*, and *Bcl2*, positions *W. ugandensis* as a potential organic inhibitor of CRC tumorigenesis. This study provides compelling evidence for its therapeutic potential, suggesting that the plant could offer significant clinical benefits in the fight against colorectal cancer.

However, while these results are promising, they also emphasize the necessity for further in vitro experimental studies involving additional cancer cell lines. in vivo models will be essential to confirm the biological relevance and therapeutic efficacy of the plant’s phytoconstituents in a living organism, where the complex interactions between the plant’s bioactive compounds and the tumor microenvironment can be fully assessed. These studies will be crucial in determining the safety, bioavailability, and optimal dosage of *W. ugandensis* extracts, as well as their potential for synergistic effects with other treatment modalities.

Moreover, targeted research into the specific metabolites in *W. ugandensis* that contribute to its modulatory effects on these apoptotic and inflammatory pathways could further elucidate its mechanism of action. In addition, future studies should incorporate a wider variety of cell lines to confirm the broader applicability of our findings, and more time- and dose-dependent studies. Such studies would help refine the plant’s pharmacological profile, leading to the identification of the most potent compounds for broader clinical application.

Despite the plant’s long history of medicinal use, *W. ugandensis* remains underexplored in the context of CRC therapy, and there is a critical need for comprehensive ethnopharmacological investigations. These studies should focus on refining extraction methods, understanding the optimal synthesis and dosage protocols, and ensuring reproducibility in therapeutic effects.

In conclusion, while the present study introduces a novel mechanistic understanding of the bioactive components of *W. ugandensis*, further in vitro *studies* are essential to validate its therapeutic efficacy. With its promising anticancer properties, *W. ugandensis* holds significant potential as an alternative to synthetic inhibitors, offering a natural, sustainable approach to colorectal cancer treatment. Therefore, advancing research into this plant could not only contribute to cancer therapeutics but also broaden our understanding of natural compounds in drug development.

## 6. Current and Potential Future Limitations of the Study

### 6.1. Current Limitations

1. Cell line specificity and generalizability:
  The use of Caco-2 cell lines, which are derived from human colorectal carcinoma, may limit the generalizability of the results to other cell types or tissues. These findings might not fully reflect the plant’s effects in other types of cells or in vivo systems.
2. Lack of in vivo validation:
  In vitro studies often do not account for the complex interactions present in whole organisms. Without in vivo validation, it is difficult to predict how the plant’s metabolites will behave in a living organism, especially considering factors like metabolism, bioavailability, and systemic effects.
3. Metabolite identification and quantification:
  The active metabolites responsible for modulating gene expression may not be fully characterized or quantified. In vitro studies may lack precise information on the specific bioactive compounds, their concentrations, and their precise mechanisms of action.
4. Dosing Challenges:
  The dosing used in in vitro studies may not directly correlate with the concentrations of the active compounds that would be achievable in human tissues or bloodstream after administration. This discrepancy can limit the relevance of findings to real-world therapeutic doses.
5. Short-Term Exposure:
  In vitro studies typically involve relatively short-term exposure of cells to treatments, which may not capture long-term effects or potential delayed responses, such as alterations in cellular pathways over time.
6. Absence of systemic interactions:
  The study may fail to account for interactions between the plant extracts and other compounds, such as drugs or endogenous molecules, that might alter or modulate the effects observed in vitro.

### 6.2. Potential Future Imitations

1. Limited understanding of plant complexity:
  *W. ugandensis* contains a complex mixture of bioactive compounds, and future studies may face challenges in isolating and characterizing each compound’s contribution to the observed effects.
  Scaling up to human studies:
2. Translating findings from Caco-2 cell lines and in vitro studies to human clinical trials poses significant challenges. Variability in human genetics, metabolism, and absorption can make it difficult to predict how the plant’s metabolites will behave in human populations.
3. Toxicity and safety profiles:
  Future studies will need to address the potential toxicity and safety profiles of *W. ugandensis* extracts. While the current study may focus on gene regulation, potential adverse effects at higher doses, chronic exposure, or long-term use need to be investigated.
4. Mechanism of action complexity:
  As more is understood about the interactions between metabolites and gene targets, new questions may arise regarding the broader pathways and networks involved. Understanding how *W. ugandensis* influences complex cellular processes beyond individual gene regulation will require advanced techniques and systems biology approaches.

## Figures and Tables

**Table 1:** Reverse and forward primer sequences adopted and applied in our experimental study.

Primer ID	Reverse Primer	Forward Primer
*COX-2*	GCAAACCGTAGATGCTCAGGGA	CGGTGAAACTCTGGCTAGACAG
*5-LOX*	CAGGTCTTCCTGCCAGTGATTC	GGAGAACCTGTTCATCAACCGC
*Bcl2*	GCCAGGAGAAATCAAACAGAGGC	ATCGCCCTGTGGATGACTGAGT
*Bcl-xL*	AACCAGCGGTTGAAGCGTTCCT	GCCACTTACCTGAATGACCACC
*Casp9*	CAACGTACCAGGAGCCACTCTT	GTTTGAGGACCTTCGACCAGCT
*HPRT1*	CTCAGGAGGAGGAAGCC	TGCTTCTCCTCAGCTTCA

## Data Availability

The datasets generated and/or analyzed during the current study are available from the corresponding author on reasonable request.
